# Quantitative assessment of liver fibrosis (qFibrosis) reveals precise outcomes in Ishak “stable” patients on anti-HBV therapy

**DOI:** 10.1038/s41598-018-21179-2

**Published:** 2018-02-14

**Authors:** Yameng Sun, Jialing Zhou, Xiaoning Wu, Yongpeng Chen, Hongxin Piao, Lungen Lu, Huiguo Ding, Yuemin Nan, Wei Jiang, Tailing Wang, Hui Liu, Xiaojuan Ou, Aileen Wee, Neil D. Theise, Jidong Jia, Hong You

**Affiliations:** 1 Liver Research Center, Beijing Key Laboratory of Translational Medicine in Liver Cirrhosis, National Clinical Research Center for Digestive Disease, Beijing Friendship Hospital, Capital Medical University, Beijing, 100050 China; 2 Department of Infectious Diseases, Nanfang Hospital, Southern Medical University, Guangzhou, 510515 China; 3 Infectious Disease Department, Affiliated Hospital of Yanbian University, Yanji, 133000 China; 4 Department of Gastroenterology and Hepatology, Shanghai General Hospital, Shanghai Jiaotong University School of Medicine, Shanghai, 200080 China; 5 Department of Gastroenterology and Hepatology, Beijing Youan Hospital, Capital Medical University, Beijing, 100069 China; 6 Department of Traditional and Western Medical Hepatology, Third Hospital of Hebei Medical University, Shijiazhuang, 050051 China; 7 Department of Gastroenterology, Zhongshan Hospital, Fudan University, Shanghai, 200032 China; 8 Pathology Department, China-Japan Friendship Hospital, Beijing, 100029 China; 9 Pathology Department, Beijing Youan Hospital, Capital Medical University, Beijing, 100069 China; 10 Department of Pathology, Yong Loo Lin School of Medicine, National University of Singapore, National University Hospital, Singapore, 119074 Singapore; 11 Department of Pathology, New York University School of Medicine, New York, NY 10016 USA

## Abstract

Current widely used semiquantitative histological assessment methods are insensitive to identify subtle changes of liver fibrosis. Therefore, to precisely assess therapeutic efficacy on chronic hepatitis B (CHB), we explored the utility of qFibrosis (a fully-quantitative morphometric method employing second harmonic generation/two photon excitation fluorescence) in liver fibrosis evaluation. Fibrosis changes were evaluated by Ishak fibrosis scoring and qFibrosis in CHB patients with paired liver biopsies before and after 78 weeks’ antiviral therapy. A total of 162 patients with qualified paired biopsies were enrolled. Ishak fibrosis scoring revealed that 42.6% (69/162) of the patients achieved fibrosis regression (≥1-point decrease), 51.9% (84/162) remained stable, and 5.5% (9/162) showed progression (≥1-point increase). qFibrosis showed similar trends in the groups of regression and progression patients as evaluated by Ishak. However, in Ishak stable patients, qFibrosis revealed hitherto undetected changes, allowing for further subcategorization into regression (*“Regression by qFibrosis”*; 40/84, 47.6%), stable (29/84, 34.5%), and progression (“*Progression by qFibrosis*”; 15/84, 17.9%) groups. These newly fine-tuned categories were supported by changes of morphological parameters of fibrosis, collagen percentage area, and liver stiffness measurements. In conclusion, qFibrosis can be used to quantitatively identify subtle changes of liver fibrosis in CHB patients after antiviral therapy.

## Introduction

There is compelling evidence that liver fibrosis and even cirrhosis can be reversed after suppression of hepatitis B virus (HBV)^1,2^. In a landmark study, treatment with tenofovir disoproxil fumarate for 5 years resulted in 51% (176/348) of patients achieving fibrosis/cirrhosis regression^3^. Similarly, the entecavir study showed 88% (50/57) of patients achieving at least a 1-point improvement in Ishak fibrosis scores after 5 years of treatment^4^.

The gold standard for evaluation of liver fibrosis improvement is still the liver biopsy^5^. Most studies have assessed fibrosis changes with semiquantitative numerical scoring systems such as stages 0 to 4 for Scheuer^6^/Metavir^7^ and 0 to 6 for Ishak system^8^. Regression has been defined as at least 1-point decrease of fibrosis stage after treatment^3,4,9^. However, in the era of potent anti-HBV treatment, relatively coarse assessment provided by the 6-point semiquantitative system may not suffice to evaluate progression or regression of fibrosis^2^.

In our recent study published in *Hepatology*, we have developed a new staging system, the *Beijing Classification*, for assessment of dynamic changes of liver fibrosis pre- and post-treatment^10^. The most important novelty of the *Beijing Classification* is that it includes not only extent (stage) of fibrosis, but the *quality* of fibrosis, namely if the specimen shows predominantly regressive, progressive or indeterminate features, the P-I-R. Although we found this classification to be particularly relevant in the era of successful antiviral therapies, we perceive a need for a more quantitative method to evaluate subtle fibrosis changes and to identify different outcomes, particularly in clinical trials.

Recently, qFibrosis, a stain-free and fully automated approach based on second harmonic generation/two-photon excitation fluorescence (SHG/TPEF) microscopy has been developed^11,12^. qFibrosis is objective and reproducible and has been demonstrated to be a reliable quantitative method for the evaluation of liver fibrosis; exhibiting high correlation with Metavir fibrosis scores^13,14^. This tool provides the potential for precise quantification of histological fibrosis changes. In this study, we aim to evaluate the finer changes in liver fibrosis by SHG/TPEF in CHB patients with paired liver biopsies performed pre- and post-antiviral treatment.

## Results

### Demographic and clinical characteristics of patients

**Figure 1:**
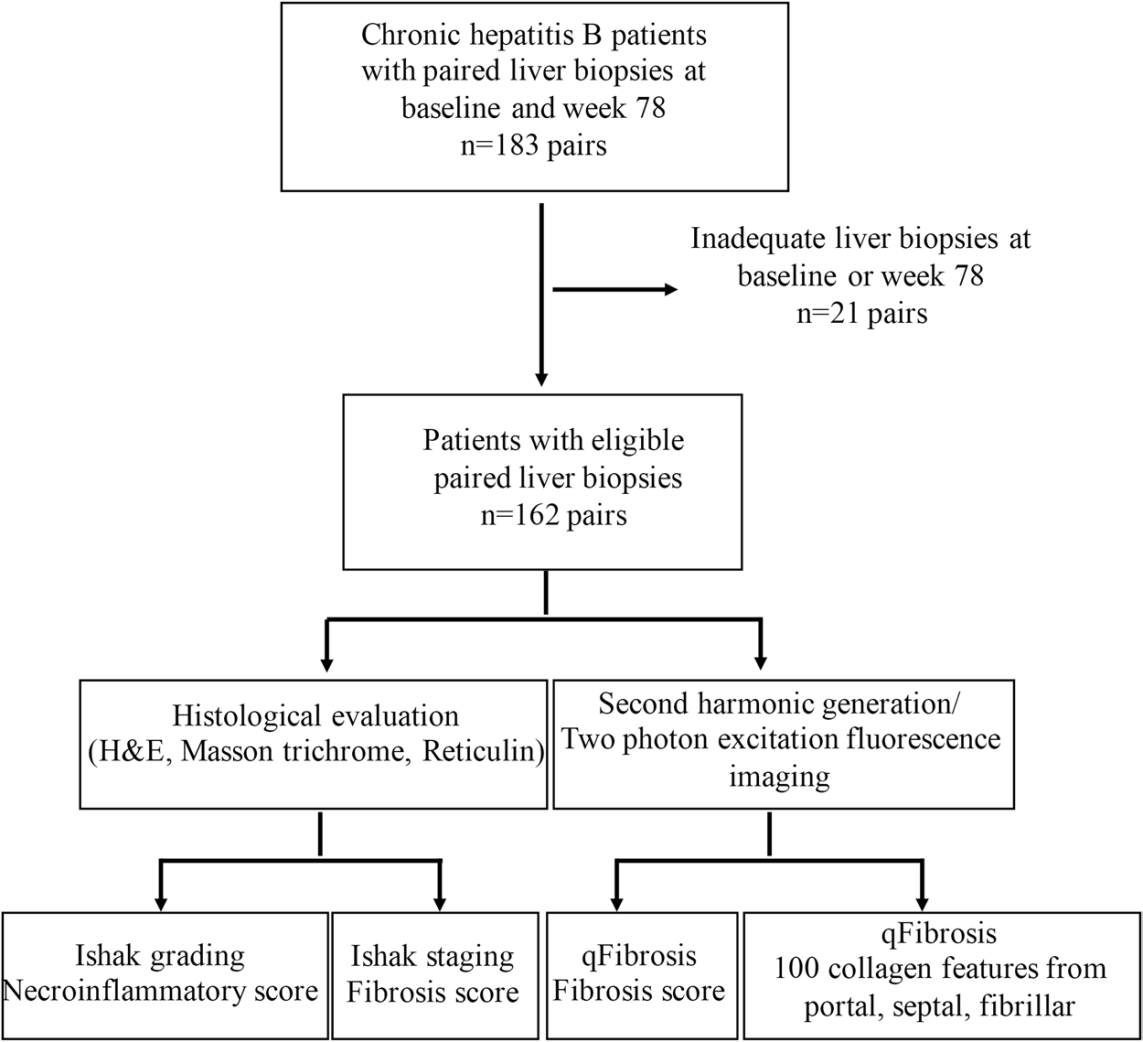
Flow chart of assessment of fibrosis regression in paired liver biopsies from chronic hepatitis B patients after antiviral therapy.

A total of 183 patients with paired liver biopsies were enrolled. After excluding unqualified biopsies, 162 patients with sufficient pre- and post-treatment liver specimens were included in the final analysis. All specimens were assessed by Ishak modified HAI grading and fibrosis staging and SHG/TPEF-based qFibrosis (Fig. 1). Patients were predominantly male (78%) with a median age of 36 years, median baseline HBV DNA of 6.80 Log IU/mL and ALT level of 81.4 U/L. After 78 weeks of antiviral therapy, 74.5% of patients had HBV DNA level <20 IU/mL, significantly decreased ALT and AST (81.4 U/L to 25.5 U/L and 52.0 U/L to 23.4 U/L, respectively; *P* < 0.001), and improved liver stiffness values (from 10.6 kpa to 6.6 kpa; *P* < 0.001) (Table 1).

**Table 1:** Characteristics of chronic hepatitis B patients with paired liver biopsies at baseline and week 78 after antiviral therapy.

Characteristics	Baseline n = 162 patients	Week 78 n = 162 patients	*P* value
Age, years	36.0 (29.0, 43.0)	—	—
Male gender, n (%)	126 (77.8)	—	—
ALT, U/L	81.4 (43.0, 147.8)	25.5 (17.1, 34.7)	<0.001
AST, U/L	52.0 (36.0, 92.1)	23.4 (19.0, 30.0)	<0.001
Albumin, g/l	43.0 (40.0, 46.0)	45.1 (43.0, 48.0)	<0.001
PTA, %	85.3 (74.9, 98.2)	102.5 (92.6, 118.0)	<0.001
INR, ratio	1.07 (1.00, 1.12)	0.99 (0.94, 1.03)	<0.001
HBV-DNA, Log IU/ml	6.80 (5.67, 7.76)	0 (0, 1.31)	<0.001
Liver stiffness value, Kpa	10.6 (7.4, 14.6)	6.6 (5.3, 8.6)	<0.001
Ishak fibrosis stage, n (%)			
0	0	3 (1.9)	<0.001
1	5 (3.1)	12 (7.4)	
2	35 (21.6)	47 (29.0)	
3	54 (33.3)	59 (36.4)	
4	43 (26.5)	23 (14.2)	
5	21 (13.0)	17 (10.5)	
6	4 (2.5)	1 (0.6)	
Necroinflammatory score, n (%)			<0.001
0–3	15 (9.3)	68 (42.0)	
4–6	68 (42.0)	91 (56.2)	
7–9	52 (32.1)	3 (1.9)	
≥10	27 (16.7)	0	
^*^P-I-R score, n (%)			<0.001
P	78 (63.9)	8 (8)	
I	31 (25.4)	23 (23)	
R	13 (10.7)	69 (69)	

ALT, alanine aminotransferase; AST, aspartate aminotransferase; PTA, prothrombin activity; INR, international normalized ratio of prothrombin time. ^*^P-I-R, predominantly progressive, indeterminate, predominantly regressive, only applicable for Ishak ≥3 so there are different case numbers pre- (n = 122) and post-treatment (n = 100).

After 78 weeks of treatment, both inflammation and fibrosis were significantly improved. The proportion of patients with pronounced necroinflammation (necroinflammatory scores ≥10) decreased from 16.7% (27/162) at baseline to 0 at week 78. In addition, the proportion of patients with advanced fibrosis (stage 4) and cirrhosis (stages 5 and 6) decreased from 42.0% (68/162) at baseline to 25.3% (41/162) at week 78. Overall, 42.6% (69/162) of patients achieved fibrosis regression (at least a 1-point improvement in Ishak fibrosis scores), 51.9% (84/162) remained stable (no change in Ishak scores), while 5.5% (9/162) showed fibrosis progression (at least a 1-point increase in Ishak scores) (Table 1).

According to P-I-R score, 63.9% (only applicable for Ishak ≥3, 78/122) of patients were predominantly progressive at baseline, but this proportion declined to 8.0% (only applicable for Ishak ≥3, 8/100) at week 78. The proportion of patients with predominantly regressive features increased from 10.7% (13/122) at baseline to 69.0% (69/100) at week 78.

### Consistency between changes in qFibrosis and Ishak scores in patients with decreased or increased Ishak stage

As shown in Supplementary Table 2, we evaluated multiple different cut-off values of ΔqFibrosis for regression. Considering both sensitivity and specificity, the range between ± 0.4 to ± 0.6 (average of ±0.5) could serve as an acceptable cut-off value. Patients were further categorized by qFibrosis into “*Regression by qFibrosis*” (≥0.5 stage decrease by qFibrosis,), “*Progression by qFibrosis*” (≥0.5 stage increase by qFibrosis), and *“Stable by qFibrosis”* (qFibrosis changes <0.5 stage).

**Figure 2:**
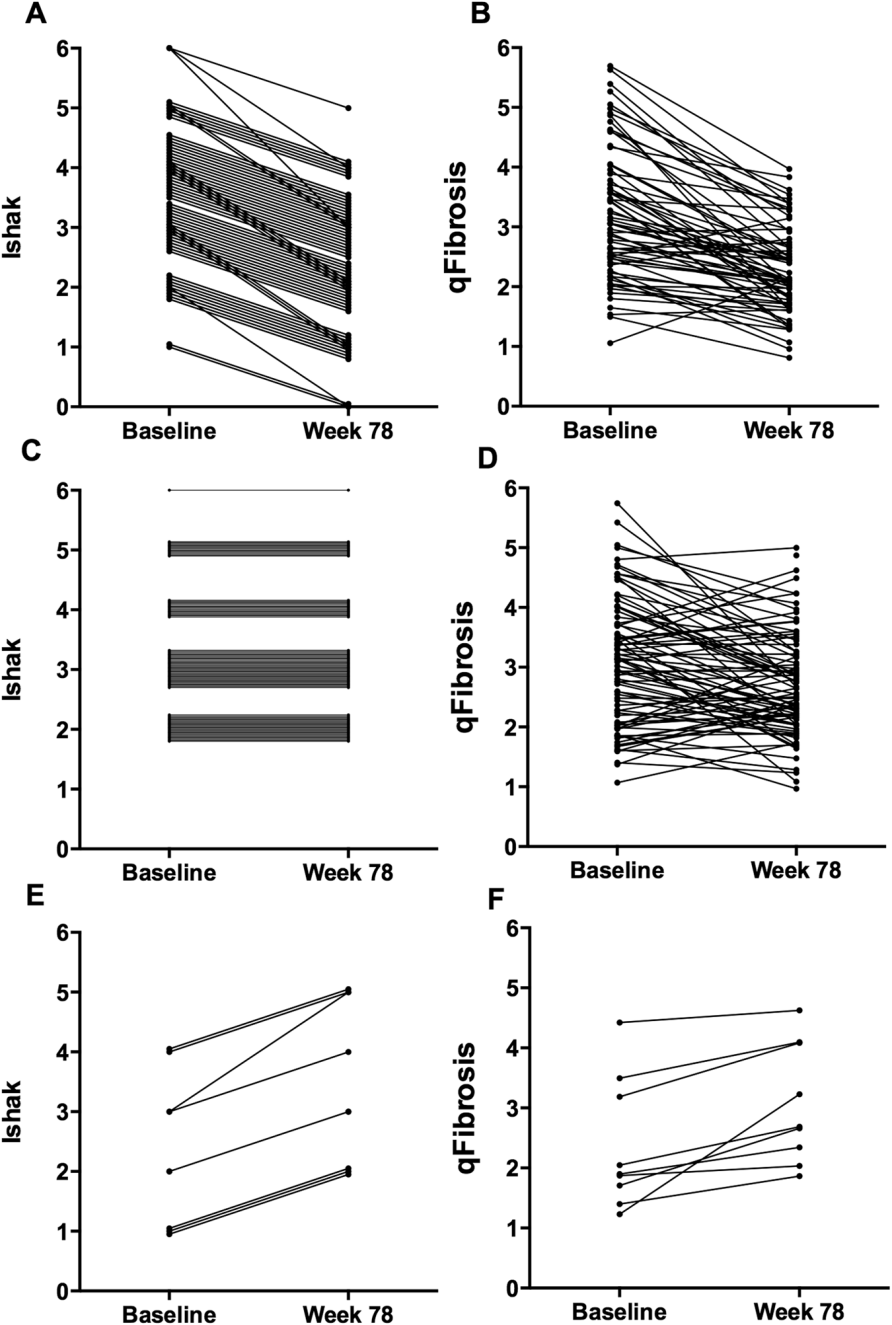
Serial changes in Ishak fibrosis scores and qFibrosis from baseline to week 78 of antiviral therapy in chronic hepatitis B patients. (**A**,**B**) Fibrosis regression (≥1-point decrease by Ishak fibrosis score), (**C**,**D**) stable by Ishak score, and (**E**,**F**) fibrosis progression (≥1-point increase by Ishak score).

There was relatively good agreement between qFibrosis and Ishak scores in patients with decreased (Fig. 2A and B) and increased Ishak stages (Fig. 2E and F). Of 12 patients with 2-stage improvements, 11 (92%) were classified as “*Regression by qFibrosis*” and one (8%) was *“Stable by qFibrosis”*. In patients with 1-stage decrease by Ishak score, 67% (38/57) were classified as “*Regression by qFibrosis*” and 30% (17/57) were considered *“Stable by qFibrosis”*. It is noteworthy that the Ishak scoring system is a categorical assignment based largely on architectural changes, instead of quantitative measurement of fibrosis content. The changes in fibrosis content between early Ishak stages in biopsy tissue are minimal; thereby lending these cases to be classified as stable by qFibrosis. In those nine progressed patients with increasing Ishak stage, 67% (6/9) were also considered *“Progression by qFibrosis”* while 33% (3/9) were reclassified as *“Stable by qFibrosis”* (Table 2).

**Table 2:** Comparison of consistency of qFibrosis with Ishak staging in paired liver biopsies from chronic hepatitis B patients after antiviral therapy.

Ishak fibrosis score Pre → Post-treatment		qFibrosis n = 162 patients			Total n = 162	Consistency (%)	Total Consistency (%)	Consistency in advanced fibrosis and cirrhosis (%)
		Regression	Stable	Progression				
Regression					n = 12		91.7	100
↓ ≥ 2 stage	2/3 → 0/1	3	1	0	4	75.0		
	4 → 2	4	0	0	4	100		
	5/6 → 3/4	4	0	0	4	100		
Regression					n = 57		66.7	82.8
↓ = 1 stage	1/2 → 0/1	4	7	0	11	36.4		
	3 → 2	10	6	1	17	58.8		
	4 → 3	17	4	1	22	77.3		
	5/6 → 4/5	7	0	0	7	100		
Stable					n = 84		34.5	13.8
No change	2 → 2	8	10	5	23	43.5		
	3 → 3	11	15	6	32	46.9		
	4 → 4	9	3	3	15	20.0		
	5/6 → 5/6	12	1	1	14	7.1		
Progression					n = 9		66.7	75.0
↑ ≥ 1 stage	1/2 → 2/3	0	2	3	5	60.0		
	3 → 4/5	0	0	2	2	100		
	4 → 5	0	1	1	2	50.0		

**Figure 3:**
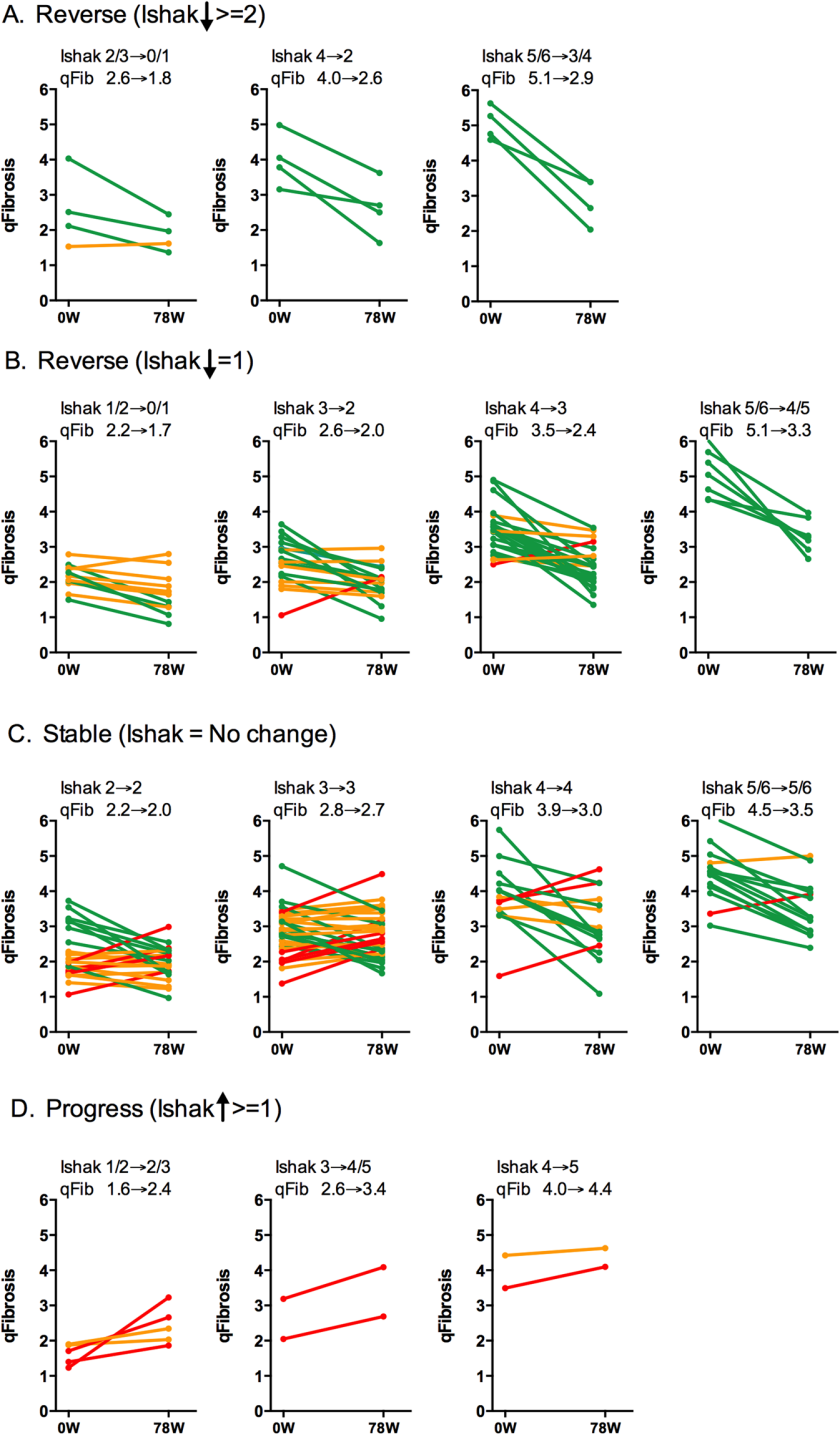
Changes in qFibrosis based on changes of Ishak fibrosis scores from baseline to week 78 of antiviral therapy in chronic hepatitis B patients. qFibrosis shows relatively good agreement with Ishak scores in patients with increased (**A**,**B**) and decreased (**D**) Ishak stages. However, in Ishak stable patients, qFibrosis can further subcategorize them into “*Regression, Stable and Progression by qFibrosis”* (**C**). Green line, “*Regression by qFibrosis*”; Red line, “*Progression by qFibrosis*”; Orange line, “*Stable by qFibrosis*”.

The more severe the fibrosis, the higher the agreement between qFibrosis and Ishak system for the regression and progression categories. Consistencies of 100%, 83% and 75%, respectively, were recorded for cases of advanced fibrosis with Ishak ≥4 (Table 2). Besides, a trend towards a greater decrease in qFibrosis was seen in those cases with a higher baseline Ishak stage. The median qFibrosis decreased by 2.2 in patients with cirrhosis at baseline, which is higher than those with advanced fibrosis (decreased by 1.4) and early fibrosis (decreased by 0.8) (Fig. 3A). A similar trend was observed in patients with 1-stage decrease in Ishak scores (Fig. 3B).

### Precise subclassification of patients with “stable” Ishak stages by qFibrosis

It was interesting to note that in those Ishak stage-stable cases, only about one third was identified as stable by qFibrosis, with the other two thirds were reassigned into either regression or progression groups by qFibrosis assessment (Fig. 2C and D; Table 2). Of 84 patients with stable Ishak scores, 34% (29/84) were *“Stable by qFibrosis”*, 48% (40/84) were “*Regression by qFibrosis*”, and 18% (15/84) were “*Progression by qFibrosis*” (Table 2). Of note is that the proportion of “*Regression by qFibrosis*” patients was higher in patients with ≥stage 4 disease than in those with Ishak stage 2–3 at baseline (Fig. 3C).

**Figure 4:**
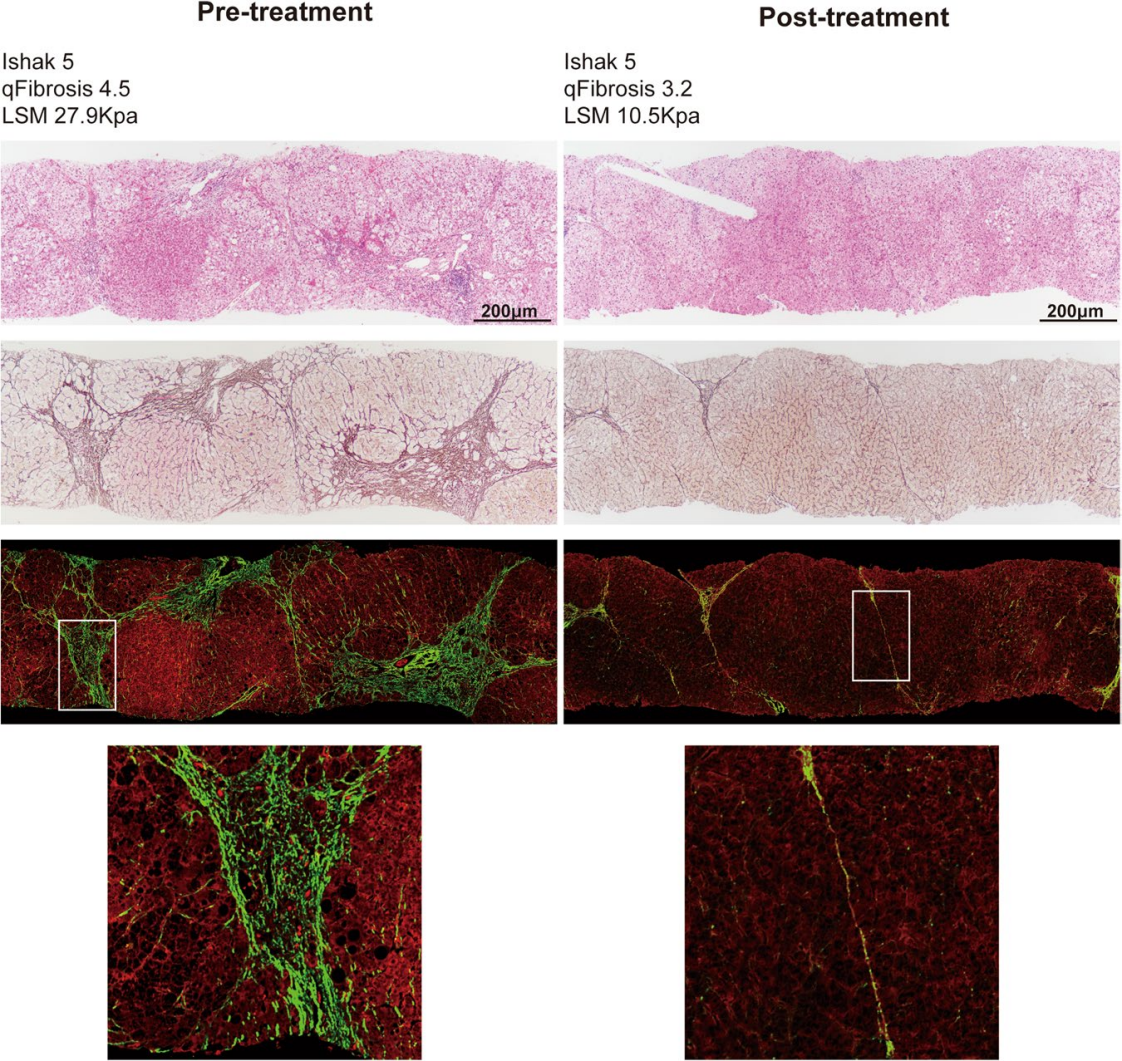
Liver biopsies with stable Ishak scores which were reassigned as *“Regression by qFibrosis”*. (**A**) Fibrous septa were wide with loosely aggregated collagen fibers and inflammatory cell infiltration at baseline. (**B**) After treatment, the septa became delicate, densely aggregated and acellular.

Figure 4 shows a representative case with paired biopsies, which were considered stable by Ishak staging but were reclassified as *“Regression by qFibrosis”*. qFibrosis decreased from 4.5 to 3.2 after 78 weeks of therapy while the Ishak scores remained at stage 5. LSM also decreased from 27.9 Kpa to 10.5 Kpa. The most remarkable regression occurred in the septal compartment. At baseline, fibrous septa were wide with loosely aggregated collagen fibers and inflammatory cell infiltrates. After treatment, the septa became delicate, densely aggregated and acellular.

### P-I-R score, collagen percentage area and liver stiffness measurement assessment to confirm subclassification by qFibrosis

Table 3 shows Ishak staging of all patients into regression, stable or progression categories. The 84 Ishak “stable” cases were reassigned as *“Regression by qFibrosis”, “Stable by qFibrosis”* and *“Progression by qFibrosis”*. To compare these new subcategories, P-I-R score was assessed. The proportion of patients with regressive fibrosis features at week 78 was 71.9% (23/32) in “*Regression by qFibrosis*” patients, higher than that in “*Stable by qFibrosis*” (57.9%, 11/19) and “*Progression by qFibrosis*” groups (60.0%, 6/10).

**Table 3:** Changes in collagen percentage areas and liver stiffness measurements between regression and progression groups by Ishak staging and qFibrosis.

	Regression by Ishak (n = 69)	Stable by Ishak (n = 84)			Progression by Ishak (n = 9)
		Regression by qFibrosis	Stable by qFibrosis	Progression by qFibrosis	
No. (%)	69 (42.6)	40 (24.7)	29 (17.9)	15 (9.3)	9 (5.6)
^**^P-I-R 78w, n (%)					
P	0	1 (3.1)	3 (15.8)	2 (20.0)	2 (33.3)
I	6 (18.2)	8 (25.0)	5 (26.3)	2 (20.0)	2 (33.3)
R	27 (81.8)	23 (71.9)	11 (57.9)	6 (60.0)	2 (33.3)
Δ LSM/Baseline, %	−44 (−63, −12)	−46 (−63, −19)	−20 (−45,+11)	−27 (−39,+1)	−17 (−36,+4)
Δ Collagen area					
Δ Total-CPA	−1.33 (−3.96, −0.42)	−2.63 (−4.31, −1.30)	−0.05 (−0.55,+0.38)	+0.74 (+0.59,+1.82)	+1.13 (+0.52,+3.30)
Δ Portal-CPA	−0.24 (−0.74, 0)	−0.17 (−0.80,+0.13)	−0.21 (−0.47,+0.18)	−0.14 (−0.40,+0.45)	+0.01 (−0.10,+2.32)
Δ Septal-CPA	−0.58 (−2.10, −0.16)	−1.31 (−2.45, −0.64)	+0.17 (−0.07,+0.44)	+0.66 (+0.53,+1.74)	+0.29 (−0.21,+1.22)
Δ Fibrillar-CPA	−0.44 (−1.24, −0.09)	−0.96 (−1.42, −0.46)	+0.04 (−0.07,+0.19)	+0.26 (+0.14,+0.57)	+0.17 (−0.05,+0.56)
			
			

^*^P < 0.05 in Δ LSM/Baseline, Total-CPA, Septal-CPA and Fibrillar-CPA; ^#^*P* > 0.05 in Δ LSM/Baseline and Portal-CPA; ^##^*P* > 0.05 in all parameters; ^**^P-I-R, predominantly progressive, indeterminate, predominantly regressive, only applicable for Ishak ≥ 3.

LSM, liver stiffness measurement; CPA, collagen percentage area.

At baseline, qFibrosis correlated well with liver stiffness (*r* = 0.6, *P* < 0.001). With the improvement of inflammation, LSM significantly decreased after treatment and the association with qFibrosis decreased (*r* = 0.3, *P* < 0.001). Changes of LSM were also evaluated. LSM decreased by 46% in “*Regression by qFibrosis*” patients, comparable to 44% in patients with decreasing Ishak stage (*P* = 0.595). Decrease of LSM in these two regression groups were more than that in “*Stable by qFibrosis*” and “*Progression by qFibrosis*” groups. Increase of LSM in “*Progression by qFibrosis*” patients was comparable with that in patients with increasing Ishak stage. Dynamic changes of LSM among groups were further assessed. As shown in Supplementary Figure 2, in the group of regression by Ishak and *“Regression by qFibrosis*” patients, LSM decreased rapidly after 26 weeks of therapy, followed by a more gradual decrease after that. However, those patients with stable or progression changes had more fluctuating liver stiffness values, especially in patients with increasing Ishak stage.

Similarly, total collagen percentage area (CPA) decreased by 2.6 in “*Regression by qFibrosis*” patients, which is even greater than the 1.3 decrease documented in patients with decreasing Ishak stage. Total CPA showed minimal change of 0.1 in “*Stable by qFibrosis*” and increase of 0.7 in “*Progression by qFibrosis*” patients. We also employed SHG/TPEF analysis to identify the three main locations of collagen. Among portal, septal and fibrillar CPA, similar results were seen in changes from septal and fibrillar compartments but not in the portal compartment, indicating a possibility that fibrosis regression may begin in septal and fibrillary areas instead of portal areas (Table 3).

We analyzed the baseline and post-treatment factors associated with fibrosis regression. As shown in Supplementary Table 3, higher baseline Ishak stage, LSM and qFibrosis were identified as predictors for fibrosis regression; this is consistent with other studies by Wang *et al*.^15^ and Marcellin *et al*.^3^.

### Changes of multi-morphological fibrosis features by Heatmap analysis to confirm subclassification by qFibrosis

**Figure 5:**
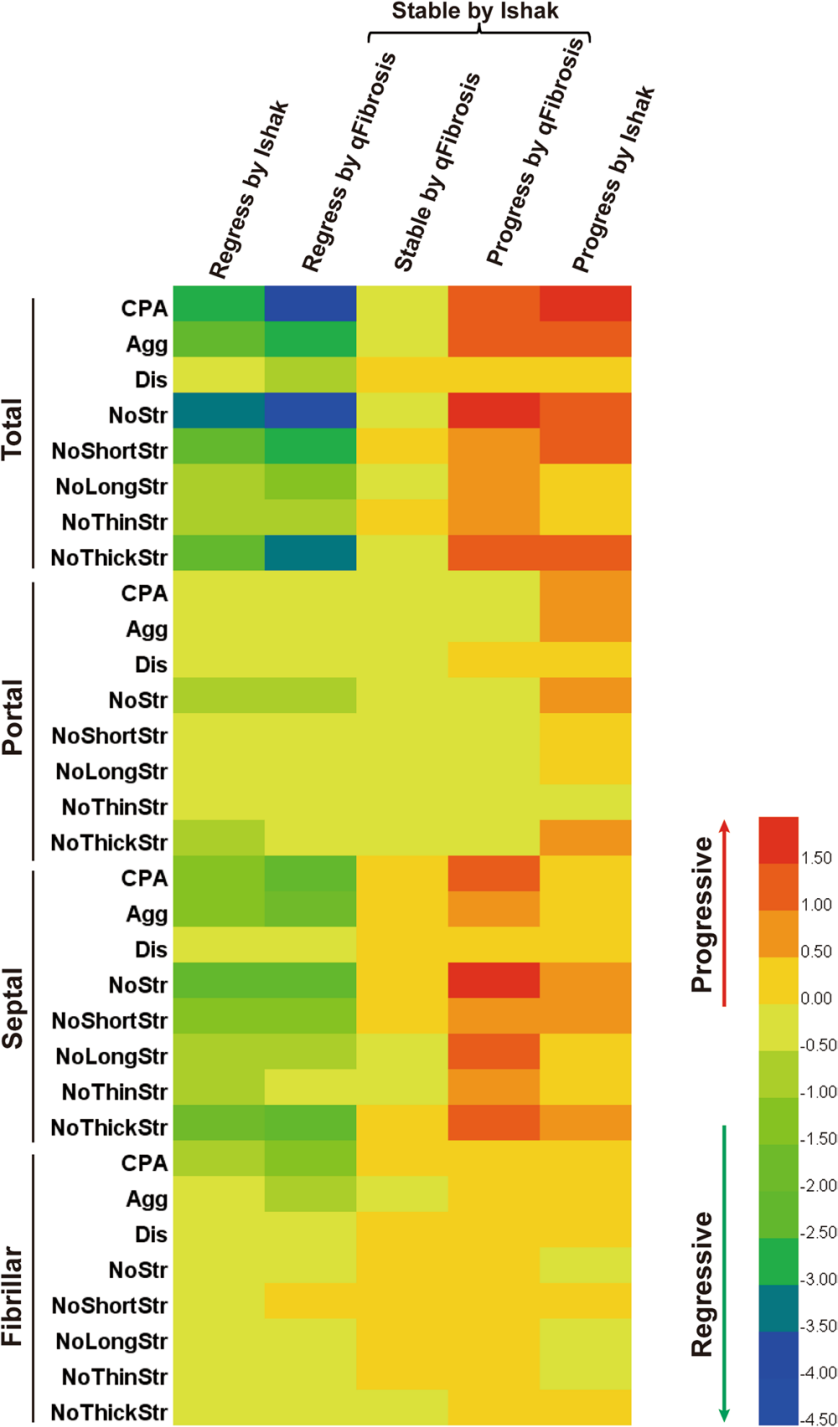
Changes of multi-morphological collagen features pre- and post-treatment by Heatmap analysis. The color scale represents the changes of collagen features ((post-pre)/pretreatment). Red color indicates fibrosis progression, whereas green or blue color indicates regression. Changes of collagen features were comparable between “*Regression by Ishak*” and “*Regression by qFibrosis*” groups. Most of these features hardly changed in *“Stable by qFibrosis*” group, but increased in “*Progression by qFibrosis*” and “*Progression by Ishak*” groups. CPA, Collagen Percentage Area; Agg, Aggregated Collagen Percentage Area; Dis, Distributed Collagen Percentage Area; NoStr, Number of Strings; NoShortStr, Number of Short Strings; NoLongStr, Number of Long Strings; NoThinStr, Number of Thin Strings; NoThickStr, Number of Thick Strings.

Multiple morphological collagen features from portal, septal and fibrillar areas were further identified by SHG/TPEF. Changes of collagen features were comparable between “*Regression by Ishak*” and “*Regression by qFibrosis*” groups (Fig. 5). Features from septal areas decreased more significantly than those from portal and fibrillar areas, supporting the rationale that the most remarkable morphological change in fibrosis regression was the thinning of fibrous septa. Most of these features changed minimally in *“Stable by qFibrosis*” group, and increased in both “*Progression by qFibrosis*” and “*Progression by Ishak*” groups.

## Discussion

In this study based on a well-characterized cohort of CHB patients on antiviral therapy with paired liver biopsies, we evaluated fibrosis regression by both semiquantitative histological fibrosis scoring (Ishak) and fully automated quantitative method (qFibrosis). Changes in qFibrosis were consistent with the increase or decrease of Ishak fibrosis scores. However, qFibrosis can further identify fibrosis improvements in patients who remained unchanged in Ishak stages.

The biggest improvement was in the analysis of those “on the fence” cases - stable as defined by Ishak; qFibrosis was able to distinguish a range of histological outcomes. In more than half (52% in this study) of the Ishak “stable” patients after anti-HBV treatment, qFibrosis identified 48% of patients actually showing significant improvement, supported by liver stiffness measurements and CPA. These complexities were previously imperceptible by the Ishak methodology alone. Even after long term antiviral therapy, some patients may still have fibrosis progression. Therefore, early detection of patients with subtle collagen increase will contribute to closer monitoring with supposedly lower incidence of clinical outcomes. Hence, qFibrosis can provide critical information on treatment evaluation and need for treatment modification, with these previously undetected changes. This precise method can also be used to assess the efficacy of new anti-fibrotic therapies in clinical trials.

Compared to conventional quantification assessment by CPA in assessing dynamic changes of fibrosis, qFibrosis provided more detailed collagen features incorporating collagen spatial location and architectural organization information^16–18^. Therefore, we evaluated changes of different collagen features from portal, septal and fibrillar areas. In this study, the new quantitative approach showed that fibrosis regression was most dominant in the septal area (Figs 4 and 5). CPA and other detailed collagen features (number of string, number of short, long, thick and thin string) from septal areas decreased significantly both in “*Regression by Ishak*” and “*Regression by qFibrosis*” groups.

Fibrous septa have been regarded as an important parameter in fibrosis progression and regression^19,20^. Some septal features (delicate, perforated and splitting) have been demonstrated to correlate with the fibrosis repair process^21^. In our previous study, we proposed a new (P-I-R) classification based on the balance between progressive and regressive septa^10^. The current study also shows the importance of septa resorption in fibrosis regression during a relatively short period of antiviral therapy; thus, implying that qFibrosis may be a useful tool to analyze the pathological mechanism of fibrosis regression.

Nowadays, non-invasive assessment methods employing either serum markers or LSM continue to be good surrogate markers for liver fibrosis evaluation in CHB patients. However, these markers have not been qualified to evaluate dynamic changes of liver fibrosis after treatment. APRI and FIB-4 have been demonstrated to be not suitable for assessment of improvements in liver fibrosis following therapy in CHB patients^22^. Besides, given the fact that most patients had decrease in ALT and hepatic necroinflammation, improvement of LSM by transient elastography and acoustic radiation force impulse imaging (ARFI) after antiviral therapy may be related to diminished necroinflammatory activity rather than fibrosis regression^23^. MR elastography (MRE) remains costly and technology-dependent for widespread use in routine practice^23^. The correlation of fibrosis improvement as predicted by non-invasive measurement with that by histology has yet to be determined. In the meantime, liver biopsy still remains the best standard to evaluate fibrosis regression following antiviral therapy in CHB patients.

Our study and this new technology have several limitations. First, fibrosis regression may improve liver-related clinical endpoints such as portal hypertension, hepatic decompensation and hepatocellular carcinoma^3^. Unfortunately, the low incidence of liver-related endpoints in our current study makes it impossible to analyze the association of complications with fibrosis regression. In our study, all patients will be followed up for another 5 years and a third liver biopsy will be performed after 3 years; the association of fibrosis regression and clinical endpoints will be evaluated then. Second, SHG/TPEF still has its limitations. The process of spatial location identification and collagen architectural features extraction should be more standardized. Thickness of tissue sections which may affect the collagen content was not considered. Architectural collagen from large portal tracts and liver capsule cannot be distinguished automatically, although we have excluded it manually. Finally, the process of feature identification and quantification is costly and time-consuming compared to the conventional semiquantitative scoring systems. Technology improvements may help to broaden its usage not only in clinical trials but in assessment of anti-fibrotic therapies in the future.

In conclusion, qFibrosis can sensitively reflect fine histological fibrosis changes and detect fibrosis improvements. This can be used in the assessment of therapeutic efficacies and fibrosis outcome evaluation in clinical trials.

## Patients and Methods

### Study population

A total of 183 CHB patients with paired liver biopsies pre- and post-antiviral treatment were enrolled in this study. Main inclusion criteria included 18 to 65 years of age; treatment- naïve status; detection of HBsAg for at least 6 months; HBV DNA levels >20,000 IU/ml for HBeAg-positive patients or 2,000 IU/ml for HBeAg-negative patients; liver biopsies performed at both baseline and week 78 after entecavir-based treatment.

Patients with history of liver decompensation (including ascites, variceal bleeding or hepatic encephalopathy) were excluded. Other exclusion criteria were as follows: presence of other chronic liver diseases; coinfection with hepatitis C or human immunodeficiency virus; alpha-fetoprotein higher than 100 ng/ml; creatinine higher than 1.5 × upper limit of normal (ULN); severe heart, lung, kidney, brain, blood diseases or other important systemic diseases; any malignant tumor; pregnant or lactating women; severe neurological or psychological disease.

The study was conducted according to the Declaration of Helsinki guidelines. The study protocol was approved by the Ethics Committee of Beijing Friendship Hospital, Capital Medical University (approval number: 2013029). All subjects gave written informed consent prior to initiating any study-specific procedures. Studies were registered at ClinicalTrials.gov (registration number NCT01938781, NCT01938820).

### Histological assessments

Liver biopsy specimens were routinely processed. Formalin-fixed, paraffin-embedded, 5 µm thick histological sections were stained with hematoxylin and eosin (H&E), Masson Trichrome and Reticulin^24^. All biopsy samples were evaluated independently by two pathologists from central laboratory. Discordant cases were further determined by a third senior pathologist (TLW). Ishak modified histology activity index (HAI) grading and staging system was employed to assess necroinflammatory activity and fibrosis^8^. P-I-R score (*predominantly progressive*, *indeterminate* and *predominantly regressive*) was used to assess the dynamic changes of liver fibrosis pre- and post-treatment^10^. All pathologists were blinded to treatment assignment, biopsy sequence and other clinical details. Fibrosis regression was defined as at least 1-point decrease in Ishak fibrosis scores.

### SHG/TPEF imaging for establishment of qFibrosis

All liver biopsy specimens were imaged by Genesis200^TM^ (HistoIndex Pte. Ltd, Singapore), employing SHG/TPEF technology-based microscopy (Supplementary Figure 1)^11,13^. Images were acquired at 20 × objective on unstained histological liver sections with 512 × 512 pixel resolution of a single 200 × 200 μm^2^ tile. In order to avoid missing information from biopsy tissues, each section was fully imaged and stitched by multiple adjacent images. To determine only disease-related collagen, image artifacts and structural collagen in large portal tracts and blood vessel were excluded. The procedure of identifying and extracting collagen features from portal, septal, and fibrillar areas can be obtained from Xu *et al*.^13^.

The sophisticated image analysis algorithms extracted 100 collagen features from SHG images. The features were then normalized by image segmentations of TPEF images which were considered as the tissue size. Correlation analysis between the collagen features and fibrosis stages and Bayesian Information Criterion (BIC) test were used to select the most important features for establishing qFibrosis. Nonlinear regression model was selected to combine the collagen features into a single index based on the distribution of fibrosis stages. Eventually, a model of 15 features (Supplementary Table 1) revealed the best performance overall. The qFibrosis was calculated by the following equation:

$$qFibrosis=\varepsilon +\sum _{i=1}^{N}({a}_{i}{X}_{i}+{b}_{i}\,\mathrm{log}({X}_{i}))$$

where *X*_*i*_ was the value of the i-th selected features. Feature selection and model fitting were processed by Matlab 2015a (The MathWorks, Inc., Natick, MA).

### Liver stiffness measurements and laboratory evaluations

Liver stiffness measured by transient elastography (Fibroscan; Echosens, Paris, France) was performed by experienced operators following the manufacturer’s recommendations^25^. The results were expressed in kilopascal (Kpa) as the median of at least 10 valid measurements. Only procedures with the success rate of at least 60% and the interquartile range-to-liver stiffness ratio lower than 30% were included in the final analysis^23^.

Liver tests (including ALT, AST, albumin and bilirubin) were performed at local center laboratories according to standard procedures. HBV serological markers (HBsAg, anti-HBs, HBeAg, anti-HBe, anti-HBc) were assessed using Abbott Architect i2000 (ABBOTT, Wiesbaden, Germany). Serum HBV DNA levels were measured by Roche COBAS^®^TaqMan^®^ HBV Test, a real-time taqman polymerase chain reaction assay which has a lower limit of quantification of 20 IU/mL.

### Statistical analysis

Categorical variables were presented as counts and percentages. Continuous variables were expressed as median (interquartile range, IQR) or mean ± standard deviation (SD). Paired samples t-test and Wilcoxon signed-rank test were used to compare clinical data pre- and post-treatment. McNemar’s test was used to compare the changes of histological scores pre- and post-treatment. One-way ANOVA and Kruskal-Wallis test were used to assess the changes of LSM and collagen percentage area (CPA) in specific conditions. All statistical tests were two-sided. Statistical significance level was set as *P* < 0.05. Data analysis were performed using SPSS (version 22.0; SPSS, Inc., Chicago, IL).

### Clinical Trial Number

NCT01938781, NCT01938820.

## Electronic supplementary material

Supplementary Information
