# Supplementary material for: Quantitative assessment of liver fibrosis (qFibrosis) reveals precise outcomes in Ishak “stable” patients on anti-HBV therapy
Source: Sci Rep. 2018 Feb 14;8:2989. doi: 10.1038/s41598-018-21179-2 (PMC5813020; doi:10.1038/s41598-018-21179-2)
Supplement: Supplementary file 1 — Supplementary Information [file 41598_2018_21179_MOESM1_ESM.pdf]

Title

Quantitative assessment of liver fibrosis (qFibrosis) reveals precise outcomes in Ishak "stable" patients on anti-HBV therapy

Authors

Yameng Sun[1], Jialing Zhou[1], Xiaoning Wu[1], Yongpeng Chen[2], Hongxin Piao[3], Lungen Lu[4], Huiguo Ding[5], Yuemin Nan[6], Wei Jiang[7], Tailing Wang[8], Hui Liu[9], Xiaojuan Ou[1], Aileen Wee[10], Neil D Theise[11], **Jidong Jia[1*], Hong You[1*]**

[*]Authors share co-corresponding authorship.

Affiliations

1. Liver Research Center, Beijing Key Laboratory of Translational Medicine in Liver Cirrhosis, National Clinical Research Center for Digestive Disease, Beijing Friendship Hospital, Capital Medical University, Beijing, 100050, China;

2. Department of Infectious Diseases, Nanfang Hospital, Southern Medical University, Guangzhou, 510515, China;

3. Infectious Disease Department, Affiliated Hospital of Yanbian University, Yanji, 133000, China;

4. Department of Gastroenterology and Hepatology, Shanghai General Hospital, Shanghai Jiaotong University School of Medicine, Shanghai, 200080, China;

5. Department of Gastroenterology and Hepatology, Beijing Youan Hospital, Capital Medical University, Beijing, 100069, China;

6. Department of Traditional and Western Medical Hepatology, Third Hospital of Hebei Medical University, Shijiazhuang, 050051, China;

7. Department of Gastroenterology, Zhongshan Hospital, Fudan University, Shanghai, 200032, China;

8. Pathology Department, China-Japan Friendship Hospital, Beijing, 100029, China;

9. Pathology Department, Beijing Youan Hospital, Capital Medical University, Beijing, 100069, China;

10. Department of Pathology, Yong Loo Lin School of Medicine, National University of Singapore, National University Hospital, Singapore, 119074, Singapore;

11. Department of Pathology, New York University School of Medicine, New York, NY, 10016, USA.

***Corresponding Author**

Hong You, MD, PhD

Jidong Jia, MD, PhD

Liver Research Center, Beijing Friendship Hospital, Capital Medical University

95 Yong-an Road, Xi-Cheng District, Beijing 100050, China

Tel: +86 010 63139019

Fax: +86 010 63038519

E-mail: youhong30@sina.com, jia_jd@ccmu.edu.cn

Supplementary Table 1. Description of 15 collagen features selected for establishing qFibrosis.

Number	Feature	Description
**1**	Total CPA	Total collagen percentage area
**2**	StrLength	String Length
**3**	StrWidth	String Width
**4**	StrSolidity	String Solidity
**5**	StrPerimeter	String Perimeter
**6**	PortalAGG	Portal aggregated collagen percentage
**7**	PortalDIS	Portal distributed collagen percentage
**8**	NoStrP	Number of Strings (Portal)
**9**	NoXlinkP	Number of Crosslinks (Portal)
**10**	NoThickStrS	Number of Thick Strings (Septal)
**11**	StrLengthS	String length (Septal)
**12**	NoXlinkS	Number of Crosslinks (Septal)
**13**	Fibrillar	Fibrillar Collagen Percentage
**14**	NoStrF	Number of Strings (Fibrillar)
**15**	NoXlinkF	Number of Crosslinks (Fibrillar)

Supplementary Table 2. ROC analysis of ΔqFibrosis for fibrosis regression

Changes of qFibrosis (post-pretreatment)	Sensitivity %	Specificity %	PPV %	NPV %
For prediction of fibrosis regression				
-0.1	88	46	55	84
-0.2	88	47	55	85
-0.3	81	50	54	78
-0.4	75	53	54	78
-0.5	71	57	55	73
-0.6	62	61	54	69
-0.7	58	65	55	67
-0.8	52	69	55	66
-0.9	46	75	58	65
-1.0	43	76	58	65

Abbreviations: PPV, positive predictive value; NPV, negative predictive value.

Supplementary Table 3 Characters associated with fibrosis regression and progression at baseline and after treatment.

	Regression by Ishak or qFibrosis	Stable by qFibrosis	Progression by Ishak or qFibrosis	*P* value
**Baseline**				
N (%)	109 (67.3)	29 (17.9)	24 (14.8)	-
Age, year	36 ± 11	36 ± 8	39 ± 9	0.444
Gender (male), n (%)	83 (76.1)	23 (79.3)	20 (83.3)	0.814
PLT, $\times10^9$/L	164.0 (123.3, 199.5)	180.0 (132.8, 229.8)	159.0 (129.8, 208.5)	0.515
ALT, U/L	87.0 (46.5, 155.8)	85.4 (37.6, 200.3)	61.5 (35.3, 98.2)	0.295
AST, U/L	53.5 (40.0, 95.0)	54.7 (32.6, 107.0)	38.5 (27.8, 55.5)	0.045
ALB, g/L	42.5 (40.0, 45.6)	43.8 (40.7, 46.8)	45.1 (40.6, 46.9)	0.097
HBV DNA, Log IU/mL	6.8 (5.7, 7.9)	6.9 (5.9, 7.7)	6.0 (4.4, 7.9)	0.300
LSM, Kpa	12.0 (8.1, 17.3)	7.5 (6.7, 11.3)	8.6 (6.8, 12.5)	0.002
qFibrosis	3.3 (2.6, 4.3)	2.6 (2.0, 3.3)	2.0 (1.7, 3.3)	<0.001
Necroinflammation score, n (%)				0.001
0-3, n=15	6 (5.5)	3 (10.3)	6 (25.0)	
4-6, n=68	41 (37.6)	15 (51.7)	12 (50.0)	
7-9, n=52	37 (33.9)	11 (37.9)	4 (16.7)	
≥10, n=27	25 (22.9)	0	2 (8.3)	
Ishak score, n (%)				0.001
1, n=5	2 (1.8)	0	3 (12.5)	
2, n=35	18 (16.5)	10 (34.5)	7 (29.2)	
3, n=54	31 (28.4)	15 (51.7)	8 (33.3)	
4, n=43	35 (32.1)	3 (10.3)	5(20.8)	

5, n=21	20 (18.3)	0	1 (4.2)	
6, n=4	3 (2.8)	1 (3.4)	0	
**Post-treatment**				
ALT, U/L	24.0 (17.0, 33.0)	28.0 (20.0, 39.0)	32.5 (16.0, 42.4)	0.230
AST, U/L	21.9 (18.0, 29.0)	27.0 (20.5, 30.5)	25.2 (21.5, 35.9)	0.048
ALB, g/L	45.6 (43.2, 48.1)	45.0 (43.0, 47.5)	44.6 (42.4, 46.3)	0.120
HBV DNA, Log IU/mL	0 (0, 1.3)	0 (0, 1.3)	0 (0, 0)	0.871
LSM, Kpa	6.3 (5.2, 8.3)	6.5 (5.3, 9.0)	6.9 (6.1, 9.6)	0.227
qFibrosis	2.3 (1.9, 2.9)	2.5 (1.9, 3.4)	2.7 (2.4, 4.0)	0.013
Necroinflammation score, n (%)				0.447
0-3, n=68	49 (45.0)	12 (41.4)	7 (29.2)	
4-6, n=91	58 (53.2)	17 (58.6)	16 (66.7)	
7-9, n=3	2 (1.8)	0	1 (4.2)	
≥10, n=0	0	0	0	
Ishak score, n (%)				0.084
0, n=3	3 (2.8)	0	0	
1, n=12	12 (11.0)	0	0	
2, n=47	29 (26.6)	10 (34.5)	8 (33.3)	
3, n=59	36 (33.0)	15 (51.7)	8 (33.3)	
4, n=23	16 (14.7)	3 (10.3)	4 (16.7)	
5, n=17	13 (11.9)	0	4 (16.7)	
6, n=1	0	1 (3.4)	0	

Figure Legend

Supplementary Figure 1. Liver biopsy samples of CHB patients by SHG/TPEF morphometry at Ishak fibrosis stages 0-6.

CHB, chronic hepatitis B; SHG/TPEF, second harmonic generation/two photon excitation fluorescence.

Supplementary Figure 2. Dynamic changes of liver stiffness measurements in groups of (A) Fibrosis regression (≥1-point decrease by Ishak fibrosis score), (B) stable by Ishak score, (C) fibrosis progression (≥1-point increase by Ishak score) and the subclassified groups (D) *Regression by qFibrosis,* (E) *Stable by qFibrosis",* (F) *Progression by qFibrosis.*

Supplementary Figure 3. Association between qFibrosis and liver stiffness pre- and post-treatment.

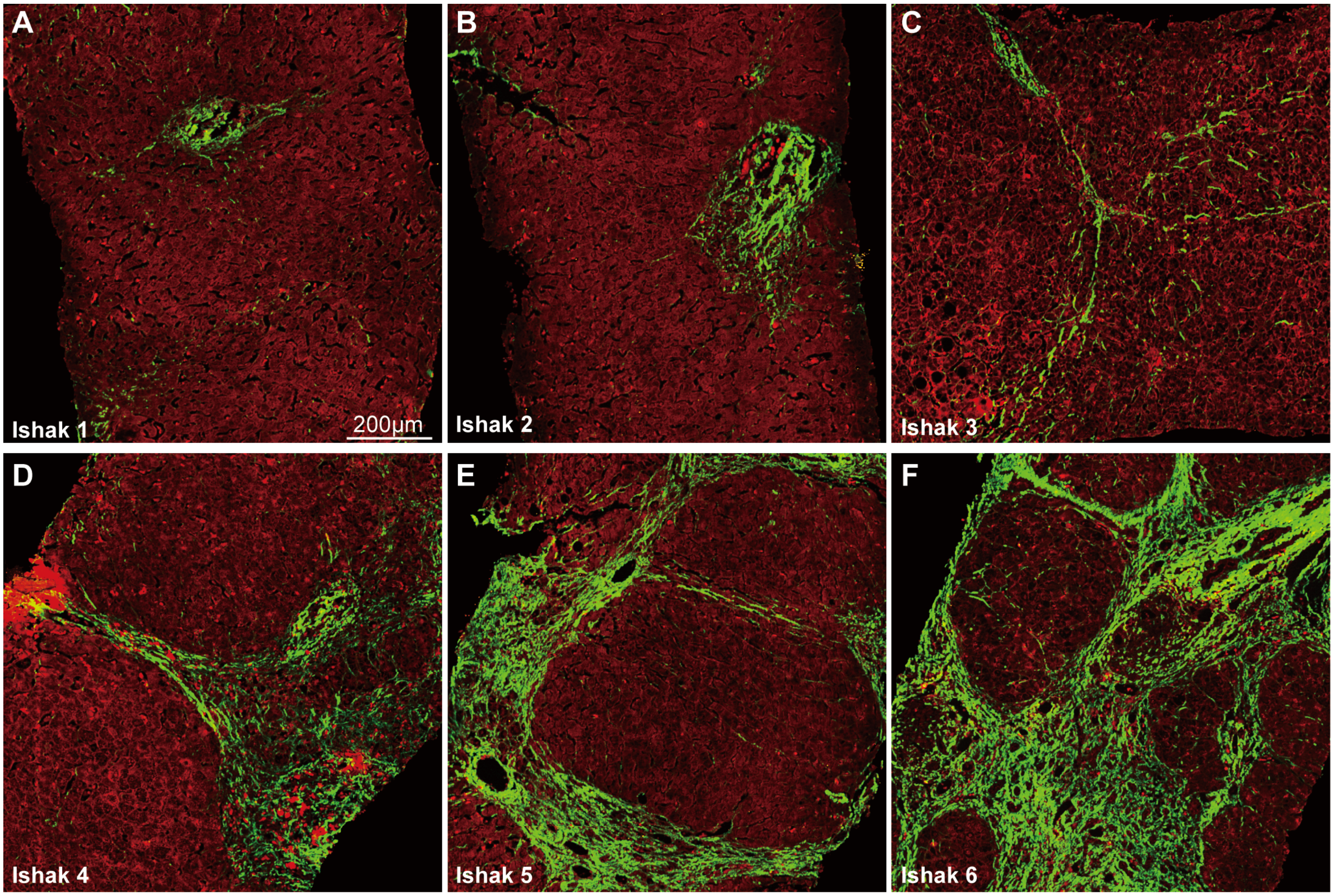
A
Ishak 1
200μm
B
Ishak 2
C
Ishak 3
D
Ishak 4
E
Ishak 5
F
Ishak 6

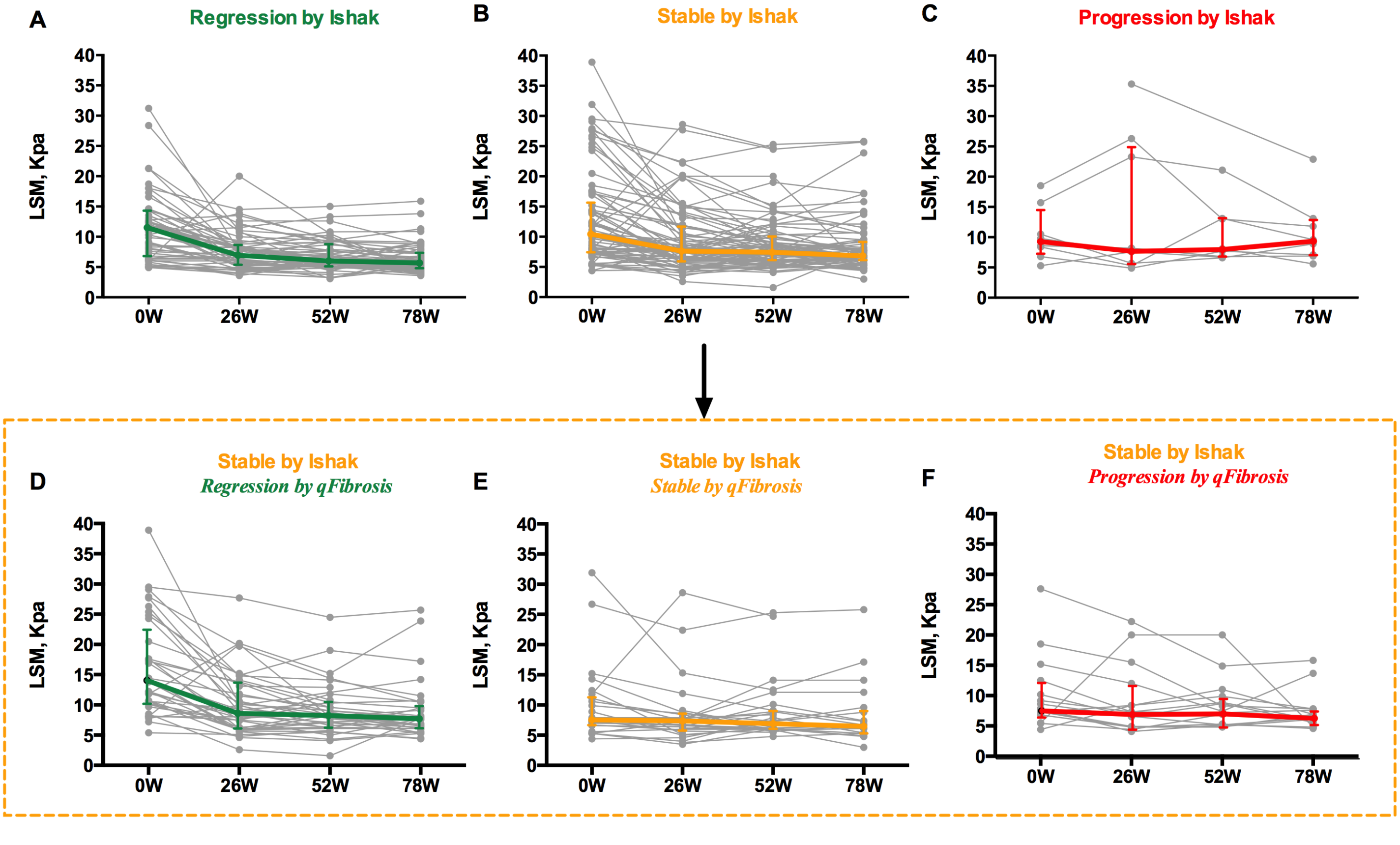
A
Regression by Ishak
B
Stable by Ishak
C
Progression by Ishak
D
Stable by Ishak
Regression by qFibrosis
E
Stable by Ishak
Stable by qFibrosis
F
Stable by Ishak
Progression by qFibrosis
LSM, Kpa
0W
26W
52W
78W
0
5
10
15
20
25
30
35
40

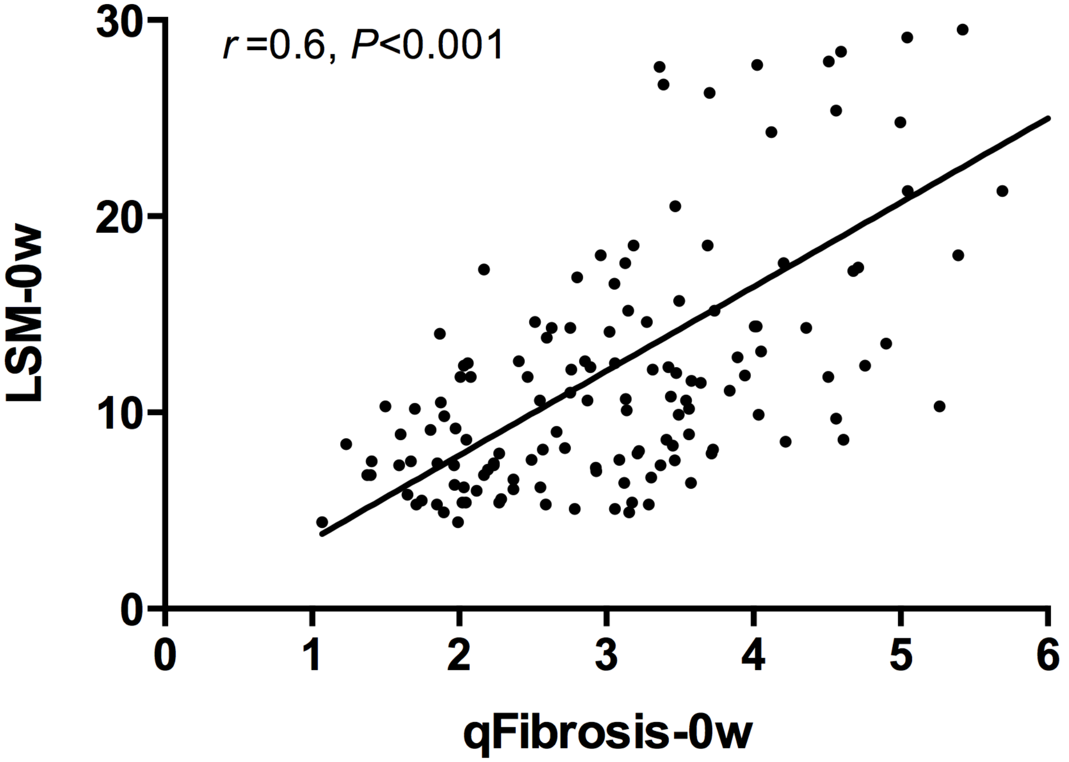

r =0.6, P<0.001
LSM-0w
qFibrosis-0w
30
20
10
0
0
1
2
3
4
5
6

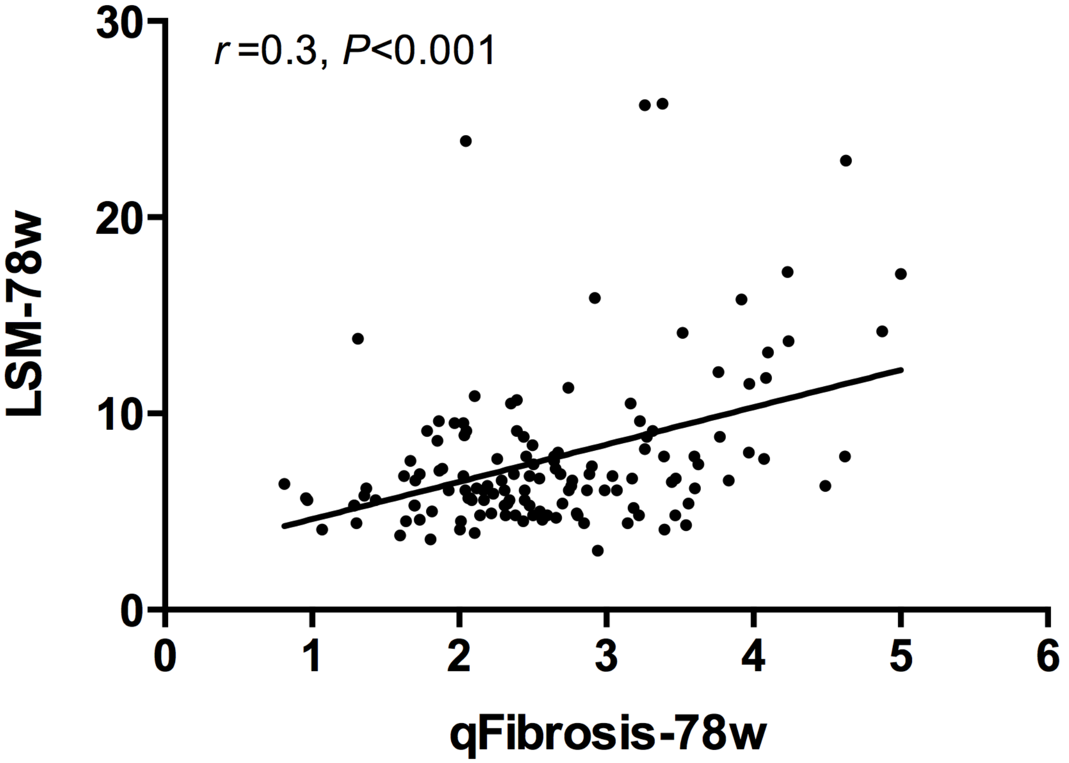

r =0.3, P<0.001
LSM-78w
qFibrosis-78w
30
20
10
0
0
1
2
3
4
5
6
